# Development of a multiplex real-time PCR assay with fluorescence probe-melting-curve analysis for one-tube detection of respiratory pathogens

**DOI:** 10.1371/journal.pone.0357377

**Published:** 2026-09-09

**Authors:** Mingyu Ji, Hongjiao Dong, Yihui Xu, Yunshan Wang, Qianqian Zhao

**Affiliations:** 1 Medical Integration and Practice Center, Shandong University, Jinan, Shandong, China; 2 Medical Research and Laboratory Diagnostic Center, Jinan Central Hospital Affiliated to Shandong First Medical University, Jinan, Shandong, China; 3 Research and Development Department, GuoRan Gene Technology (Shandong) Company Limited, Jinan, Shandong, China; 4 Shandong LaiBo Biotechnology Company Limited, Jinan, Shandong, China; International Center For Chemical and Biological Sciences, PAKISTAN

## Abstract

Pathogens causing respiratory infections have reemerged globally since the COVID-19 pandemic. Rapid and accurate identification of respiratory pathogens is critical for diagnosis and treatment. In this study, we developed and evaluated a multiplex real-time PCR assay with a fluorescence probe melting curve (mqPCR-PMC) assay to simultaneously detect six common pathogens, namely, influenza virus A and B (IFV-A and -B), respiratory syncytial virus (RSV), human rhinovirus (HRV), human adenovirus (HAdV) and *Mycoplasma pneumoniae* (MP). The primers and probes optimized in this assay exhibited a specificity of 100% with a panel of 57 pathogens. The detection limits of the assay ranged from 248 copies/ml to 394 copies/ml. Moreover, the coefficients of variation ranged from 0.11% to 0.30% for Tm and from 1.59% to 4.10% for Rm. The clinical accuracy of the assay was validated using 760 pharyngeal samples. Compared with a commercial kit based on conventional fluorescence RT-PCR, the assay showed more than 87% sensitivity and 99% specificity for each pathogen. The kappa values ranged from 0.927 to 1.000. Moreover, the assay was more precise for HRV and more sensitive on IFV-A, HAdV, MP and RSV. In summary, the mqPCR-PMC assay was demonstrated to be highly sensitive, highly specific and high throughput, and has potential for clinical use in respiratory infectious disease diagnosis.

## Introduction

Respiratory tract infections (RTIs) impose a large health care burden because of high morbidity and mortality [1,2]. They occur frequently in people of all ages with approximately 4 million fatalities every year [3]. RTIs can be caused by several different pathogens, such as viruses, bacteria, fungi, mycoplasma, or chlamydia. In addition to severe acute respiratory syndrome coronavirus 2 (SARS-CoV-2), the most commonly involved pathogens have included influenza virus A (IFV-A), influenza virus B (IFV-B), respiratory syncytial virus (RSV), human rhinovirus (HRV), human adenovirus (HAdV), and *Mycoplasma pneumoniae* (MP) [4,5]. Between 2021 and 2023, during the COVID-19 pandemic, among viruses other than SARS-CoV-2, HRV and enterovirus were reported as the most common viruses among children with acute respiratory infection (26.1%), upper respiratory tract infection (21.0%), pneumonia (97.3%), and severe acute respiratory infection (54.7%) [6]. In mainland China, HAdV infections account for 5.8% to 13% of patients with acute respiratory infections [7]. MP is responsible for approximately 40% of community-acquired pneumonia cases [5]. Moreover, co-infections were detected in different age groups, patients with different diseases, and between different pathogens. A total of 12.4% of patients with novel coronavirus pneumonia were co-infected with other respiratory viruses, such as influenza virus, RSV, HAdV, MP, Chlamydia pneumoniae (CP), and *Legionella pneumophila* (LP), of whom 5.2% had multiple infections [8]. A patient from Cuba was found to be co-infected with 16 pathogens [9]. Co-infection significantly increases the disease severity, further complicating clinical management [10,11].

These pathogens usually lead to a range of similar symptoms, including fever, mild cough, pharyngalgia, and runny nose, which so-called as “febrile respiratory syndrome (FRS)” [12]. Therefore, the etiological identification of FRS largely depends on rapid and accurate laboratory detection. Nucleic acid detection proved highly accurate and convenient during the novel coronavirus pandemic, and it has gradually replaced the conventional culture-based method to become the gold standard for pathogen diagnosis [13–15]. Many technologies for nucleic acid detection have been established such as real-time polymerase chain reaction (qPCR), digital PCR (dPCR), isothermal amplification technology, and targeted and meta-genomic next-generation sequencing (tNGS and mNGS, respectively) [16–19]. Among them, qPCR-based commercial kits are the most commonly used because of their simple operation and low cost [20]. Multiplex amplification enables the simultaneous detection of several pathogens, using distinct pairs of oligonucleotide primers and probes. Although the fluorescent signal is typically used as a marker due to its high sensitivity and low susceptibility to interference from biological molecules, multiplexing is typically constrained by the number of fluorescence detection channels (usually 4–6), with most conventional designs assigning one target per channel. Increasing the number of reaction tubes is the method adopted by most commercial kits to detect more pathogens, which is inconvenient for FRS diagnosis. Other methods that exploit additional dimensions, such as probe melting temperature or combinatorial labeling have been reported to extend multiplexing capacity [21,22].

Here, this study aimed to conveniently detect more pathogen targets in one tube. We developed a multiplex qPCR assay combined with a fluorescence probe melting curve (mqPCR-PMC) assay to simultaneously detect six common respiratory pathogens: IFV-A, IFV-B, RSV, HRV, HAdV, and MP. This assay identified different pathogens in four detection channels using a fluorescent group at the 5’ end and the melting temperature (Tm) of each specific probe. The usefulness of the assay was evaluated by comparison with that of a commercial kit using throat swabs.

## Materials and methods

### Design of primers and probes

The complete genome sequences were used for primer and probe design for each pathogen. Published genome sequences for each pathogen were obtained from the GenBank database (https://www.ncbi.nlm.nih.gov/) and aligned using CLUSTAL W software (http://www.ebi.ac.uk/clustalw). Genotypes H1N1, H3N2, H5N1, and H7N9 were included in addition to general types of IFV-A. To design the HAdV primers, species B types (B3, B7, and B55), C types (C1, C2, and C5), and E4 types, which are associated with severe respiratory disease infections, were selected [23–27].

Specific primers were screened on the basis of conserved regions to make the amplicon less than 200 bp. The probe sequence and length were designed to ensure that different T_m_s were used for different pathogens. In addition, to divide them into four detection channels, the probes were labeled with different fluorophores and quenchers. The specificity of the primer and probe sequences was analyzed using BLAST (http://blast.ncbi.nlm.nih.gov/Blast.cgi). Additionally, the human ribonuclease P (RNaseP) gene was selected as an internal control (IC) for nucleic acid extraction and the mqPCR-PMC assay. The details of the primers and probes are shown in Table 1. All the primers and probes were synthesized by Sangon Biotech Co., Ltd. (Shanghai, China).

**Table 1:** Characteristics of primers and probes used in mqPCR-PMC analysis.

Pathogen	Groups	Target gene^a^	Primer or Probe	Sequence (5’-3’)	Aplicon Size (bp)	Melting temperature (Tm,°C)
IFV-A	g1	M1	IFV-A-F1	GGARTGGMTAAAGACAAGACC	105	
			IIFV-A-R1	CGTCTACGCTGCAGTCC		
			IFV-A-P1	FAM-TTCACGCTCACCGTGCCC-BHQ1		71.12
	g2	M1	IFV-A-F2	TTCTAACCGAGGTCGAAACG	123	
			IFV-A-R2	CCATGAGAGCCTCRAGATC		
			IFV-A-P2	FAM-GCCCCCTCAAAGCCGAGAT-BHQ1		69.66
	g3	PB1	IIFV-A-F3	ATGGTGTCTAGGGCCCG	122	
			IFV-A-R3	TATTTTTGCCGTCTGAGYTC		
			IFV-A-P3	FAM-GATCATGAAGATCTGTTCCACCATTG-BHQ1		68.26
IFV-B	g1	NP	IFV-B-F1	GCTCTAAGCAAGCAAGCT	101	
			IFV-B-R1	ATTGAGTCATTCATCATCTTGAG		
			IFV-B-P1	HEX-TGTCAATGAATATTGAGGG-BHQ1		55.68
	g2	NS1	IFV-B-F2	ATGGCCATCGGATCCTCA	121	
			IFV-B-R2	GATAATCGGTGCTCTTGACC		
			IFV-B-P2	HEX-TGAAGGACATTCAAAGCC-BHQ1		58.67
	g3	PA	IFV-B-F3	TTATTACAAGAAACTTCCAGACTAC	100	
			IFV-B-R3	TTGAATAGCATTGCTGGTTG		
			IFV-B-P3	HEX-CACAATGGCAGAATTTAGTGA-BHQ1		60.14
HRV	g1	5’ UTR	HRVA-F1	GGTGTGAAGAGCCSCG	129	
			HRVA-R1	TYGGTCCCATCCCGC		
			HRVB-F1	GACATGGTGTGAAGGCTCG	141	
			HRVB-R1	AAGTAGTCGGTCCCRTCC		
			HRVC-F1	TGAAGAGCCTATTGTGCTCACTT	134	
			HRVC-R1	CCAAAGTAGTTGGTTCCATCC		
			HRV-P1	ROX-CATTTCCTCCGGCCCCTGAATGAATG-BHQ2		76.98
	g2	5’ UTR	HRVA-F2	GGGTGTGAAGAGCCSCGT	151	
			HRVA-R2	GAAACACGGACACCCAAAGTA		
			HRVB-F2	GACATGGTGTGAAGACTCG	141	
			HRVB-R2	AAGTAGTCGGTCCCRTCC		
			HRVC-F2	GACAGGGTGAGAAGGCC	143	
			HRVC-R2	CCAAAGTAGTTGGTTCCATCC		
			HRV-P2	ROX-ATGAGTCCTCCGGCCCCTGACTCAT-BHQ2		75.89
HAdV	g1	E2B	AdV-F1	TGATGCGTTTCTTACCTYTGG	179	
			AdV-R1	CTTYGTGCTGGCYTGGAC		
			AdV-P1	ROX-CAATAGGCTGTCCGTGTCCCCGTACTATTG-BHQ2		75.06
	g2	E2B	AdV-F2	GGTCAAAAACMAGKTTTCC	102	
			AdV-R2	GGGACACGGACAGCC		
			AdV-P2	ROX-CCATTTTTGATGCGTTTCTTACTGG-BHQ2		67.8
*MP*	g1	DNase	MP-F1	GTTCTGAAAGAACATTTCCGC	141	
			MP-R1	CACGTCCTTCATCGCCTA		
			MP-P1	ROX-TTCTTTCAAAACTGAAAACGACAATCTTTCTAGTTCCAAATA-BHQ2		57.33
	g2	DNase	MP-F2	GGAACAGGCCAAACTTATCA	108	
			MP-R2	GGGGCTATCACCCTTTTG		
			MP-P2	ROX-AGATAGGGGTTGTAGGGCTTGCAATGTG-BHQ2		73.02
	g3	mgpC	MP-F3	TACCGCTAGGGACGTTGTCACT	86	
			MP-R3	CGGTGTCGTAAAGGGCGTGTA		
			MP-P3	ROX-CACCTACCTCCTCCAAGACCACAACACCCTCACC-BHQ2		80.52
RSV	g1	L	RSV-F1	TACTAATTTAGCTGGACATTGGATTC	117	
			RSV-R1	TTCAAATTAATGAACATATGATCAGT		
			RSV-P1	CY5-GGTATTTTTGAAAAAGATTGGGGAGAGG-BHQ3		69.14
	g2	F	RSVA-F2	GTGTAACKACACCTKTAAGCAC	151	
			RSVA-R2	ATTATWGACATGATAGARTAACTTTG		
			RSV-P2	CY5-TCATTAATCAATGATATGCCTATAACAAATGATC-BHQ3		67.11
Internal control		RnaseP	IC-F1	GCCATTTGGCGCTAATGTTC	188	
			IC-R1	CCTGCAGAGTGAACGTGTTA		
			IC-P1	CY5-GATCCTGTAAAATAAGTTTTCCTAGGTGTAGTTTGTC-BHQ3		58.09

^a^Matrix protein 1(M1) and polymerase PB1 (PB1) from IFV-A; nucleoprotein (NP), nonstructural protein 1 (NS1) and polymerase PA (PA) from IFV-B; 5'Untranslated Region (5'UTR) from HRV; DNA polymerase (E2B) from HAdV; Deoxyribon uclease (DNase) and MgpC family cytadherence protein (mgpC) from MP; RNA-dependent RNA polymerase (L) and fusion glycoprotein (F) from RSV

### Development of the mqPCR-PMC assay

The reaction mixture (50 μl) contained 2 μl of primer and probe mix, 16 μl of reaction buffer(50 mM Tris-HCl, 200 μM dNTPs/dUTP, 50 mM KCl and 3.0 mM MgCl_2_), 4 μl of enzyme mix (hot start DNA polymerase, reverse transcriptase, UDG enzyme and RNase inhibitor; AccurSTART U + One Step RT-qPCR Probe Kit (FOR FAST), Q231,Vazyme biotech,Nanjing, China), and 28 μl of nucleic acid template (1 ng/μl). In the group optimization of the primers and probes, 50 nM forward primer, 250 nM reverse primer, and 40 nM probe were used in the reaction mixture. UDG was used to remove the contamination of amplified products. Afterward, the concentrations of the primers and probes were optimized by a triple-factor orthogonal test, and the concentration of the forward and reverse primers ranged from 40 to 105 nM and 250–490 nM, respectively, at ratios of 1/5, 1/6, and 1/7, while the concentrations of the probes ranged from 40 to 105 nM (S1 Table). The melting curve peak area and Tm were observed and analyzed. The reaction reagents for optimization were purchased from Vazyme Biotech (Nanjing, China).

The assay was performed on a SLAN-96S automatic fluorescence PCR analysis system using the following conditions: initial reverse transcription and denaturation (55 °C for 15 min and 95 °C for 3 min); 50 cycles of PCR amplification (95 °C for 10 sec, 55 °C for 20 sec, and 72 °C for 20 sec); and a final melting stage (95 °C for 10 sec, 50 °C for 1 min, and rising from 50 °C to 90 °C at a speed of 0.2 °C/sec). Fluorescence readings were taken during the last heating increase process. The melting peak was analyzed in different fluorescence detection channels according to the fluorescent group at the 5’ end of the probe (Table 1). Simultaneously, the height of the melting peak (Rm value), which was automatically calculated by the software, was recorded. A positive result was defined as the appearance of a melting peak at the corresponding Tm. And Rm value ≥ 5 was used as the cutoff value for an effective melting peak across all the fluorescence channels, on the basis of the distributions of 37 negative controls and 10 non-target samples (S1 Fig).

### Performance of the mqPCR-PMC assay

The specificity of mqPCR-PMC was evaluated by testing nucleic acids from 57 quality control or reference panels of viruses, bacteria, fungi, MP and CP (Table 2). The types of pathogens were mainly considered in the following aspects: i) common subtypes for respiratory pathogen detection by the kit; ii) other microorganisms in the respiratory tract samples of infected people; and iii) other pathogens that might have appeared in the samples. The quality control or reference substances were purchased from BDS Biotech (Guangzhou, China) and BNCC (Henan, China). Nucleic acid from each of the 57 pathogens was extracted according to the manufacturer’s instructions using a nucleic acid extraction and purification kit (magnetic bead method, Zybio, Chongqing, China) and detected under optmized mqPCR-PMC assay conditions. The detection results were recorded and compared with the theoretical results. Excellent specificity could be demonstrated when the two results were consistent, and no cross-reaction could be judged if negative results were obtained for non-target pathogens.

**Table 2:** Quality control or reference panel used in specificity evalution of mqPCR-PMC assay.

Pathogens	Detection result					
	IFV-A	IFV-B	HRV	HAdV	MP	RSV
**Quality control**	+	+	+	+	+	+
**IFV-A HINI**	+	−	−	−	−	−
**IFV-A H7N9**	+	−	−	−	−	−
**IFV-A H3N2**	+	−	−	−	−	−
**IFV-A H5N1**	+	−	−	−	−	−
**IFV-A H1N1(2009)**	+	−	−	−	−	−
**IFV-B-Yamagata**	−	+	−	−	−	−
**IFV-B-Victoria**	−	+	−	−	−	−
**HRV-A**	−	−	+	−	−	−
**HRV-B**	−	−	+	−	−	−
**HRV-C**	−	−	+	−	−	−
**HAdV-C1**	−	−	−	+	−	−
**HAdV-C2**	−	−	−	+	−	−
**HAdV-B3**	−	−	−	+	−	−
**HAdV-E4**	−	−	−	+	−	−
**HAdV-C5**	−	−	−	+	−	−
**HAdV-B7**	−	−	−	+	−	−
**HAdV-B55**	−	−	−	+	−	−
**MP**	−	−	−	−	+	−
**RSV-A**	−	−	−	−	−	+
**RSV-B**	−	−	−	−	−	+
**CP**	−	−	−	−	−	−
**Measles virus**	−	−	−	−	−	−
**Mumps virus**	−	−	−	−	−	−
**Rubella virus**	−	−	−	−	−	−
**human Metapneumovirus**	−	−	−	−	−	−
**Coronavirus-229E**	−	−	−	−	−	−
**human Parainfluenza virus type 1**	−	−	−	−	−	−
**human Parainfluenza virus type 2**	−	−	−	−	−	−
**human Parainfluenza virus type 3**	−	−	−	−	−	−
**human Bocavirus**	−	−	−	−	−	−
**Enterovirus type 71**	−	−	−	−	−	−
**Coxsackie virus A**	−	−	−	−	−	−
**Cytomegalo virus**	−	−	−	−	−	−
**Epstein-Barr virus**	−	−	−	−	−	−
**Herpes simplex virus-1**	−	−	−	−	−	−
**Varicella-Zoster Virus**	−	−	−	−	−	−
**Staphylococcus aureus**	−	−	−	−	−	−
**Staphylococcus epidermidis**	−	−	−	−	−	−
**Pseudomonas aeruginosa**	−	−	−	−	−	−
**Escherichia coli**	−	−	−	−	−	−
**Bordetella pertussis**	−	−	−	−	−	−
**Haemophilus influenzae**	−	−	−	−	−	−
**Mycobacterium tuberculosis**	−	−	−	−	−	−
**Streptococcus salivarius**	−	−	−	−	−	−
**Streptococcus pneumoniae**	−	−	−	−	−	−
**Streptococcus pyogenes**	−	−	−	−	−	−
**Neisseria meningitidis**	−	−	−	−	−	−
**Legionella pneumophila**	−	−	−	−	−	−
**Corynebacterium diphtheriae**	−	−	−	−	−	−
**Lactobacillus bulgaricus**	−	−	−	−	−	−
**Moraxella catarrhalis**	−	−	−	−	−	−
**Klebsiella pneumoniae**	−	−	−	−	−	−
**Pneumocystis jiroveci**	−	−	−	−	−	−
**Candida albicans**	−	−	−	−	−	−
**Cryptococcus neoformans**	−	−	−	−	−	−
**Aspergillus fumigatus**	−	−	−	−	−	−
**Aspergillus flavus**	−	−	−	−	−	−

The sensitivity of the mqPCR-PMC assay was determined using dilutions of nucleic acid reference materials (RMs) (BNCC, Henan, China). Initial concentration of 2000 copies/ml were prepared using nucleic acid RMs of IFV-A-H3N2, IFV-B-Yamagata, RSV-A, HAdV-C1, HAdV-C2, HAdV-E4, HAdV-C5, HAdV-B7, HAdV-B55, HRV, and MP. Subsequently, 2-fold serial dilutions were prepared with negative pharyngeal swab eluate, corresponding to concentrations ranging from 2000 to 0 copies/ml. To inspect the amplification, fluorescence readings were added at 72 °C for 20 sec during the 50 cycles of PCR amplification. For each target, every concentration was repeated in 20 wells, and Simple logistic regression was applied to determine the limit of detection (LoD), which was defined as the minimum concentration with a positive detection rate of 95%. In the simple logistic regression, concentration was used as the predictor. The model was fitted using individual replicate outcomes at each concentration, in which each detection result was statistically analyzed through binary classification.

The repeatability of the mqPCR-PMC assay was evaluated using nucleic acid from quality controls of IFV-A-H7N9, IFV-B-Victoria, RSV-A, HAdV-C1, HRV and MP (BNCC, Henan, China). Nucleic acid was extracted, and 5 repetitions were performed. The Tm and Rm values were recorded to calculate the coefficient of variation (CV).

### Clinical evaluation of the mqPCR-PMC assay

The clinical performance of the mqPCR-PMC assay was verified by detecting pharyngeal samples from patients with typical symptoms of respiratory infections (fever, mild cough, pharyngalgia, and runny nose). A total of 760 suspected patients were recruited from January 6, 2024, to March 10, 2025, at Jinan Central Hospital Affiliated to Shandong First Medical University. To accumulate as many positive cases for each pathogen target as possible, sample collections were preferred in winter and spring seasons or during the epidemic period of specific pathogens. Pharyngeal secretions were collected using nylon swabs and immersed in 3 ml of viral transport medium (Babio, Jinan, China). Two hundred microliters of each sample was subjected to nucleic acid extraction using a nucleic acid extraction and purification kit (Zybio, Chongqing, China) and detected by mqPCR-PMC. For all the samples, the IC target should be successfully amplified and generate an effective melting peak (Rm value ≥ 5) at the corresponding Tm. Samples with failed IC amplification should be retested. If the retesting was still invalid, resampling was necessary. Simultaneously, a commercial kit (Sansure, Hunan, China)based on conventional real-time RT-PCR technology was used as the reference reagent to detect the six pathogens described above. The pathogen results of the two methods were classified to perform paired Wilcoxon Signed-Rank Test according to the description by Kuang et al [18]. On the basis of on the reference conclusions, the positive percent agreement (PPA), negative percent agreement (NPA), sensitivity, specificity,positive predictive value (PPV), negative predictive value (NPV) and 95% confidence interval (CI) were calculated. Pathogens with discrepancies were further confirmed through tNGS.

### tNGS sequencing

All inconsistent pharyngeal samples were sent to Pathogeno Medical Laboratory (Jinan, China) for tNGS sequencing. The detection panel covered a spectrum of 288 microorganisms, including 24 DNA viruses, 58 RNA viruses, 112 bacteria, 61 fungi, 3 chlamydiae, 3 mycoplasmas, 12 parasites and 66 others. The panel covered the six pathogens and subtypes used in the mqPCR-PMC assay and the reference reagent.

### Ethics statements

This research was approved by the Institutional Review Board of Shandong First Medical University (IRB No. R202303060105). Written informed consent was obtained from all participants, and written parental consent was obtained from the minors before the sample was collected.

### Statistical analysis

Statistical analysis was performed using GraphPad Prism 10 software. Measurement data are shown as the mean ± standard deviation (x ± s). Descriptive statistics of the column analysis were used to summarize the mean, standard deviation (SD), and coefficient of variation (CV). Cohen’s kappa values with 95% CIs were calculated using GraphPad QuickCalcs (https://www.graphpad.com/quickcalcs/kappa1/) (GraphPad).

## Results

### Primer and probe optimization of the mqPCR-PMC assay

For each pathogen, according to the multiple sequence alignment results, two or more groups of primers and probes were designed at the gene as follows: matrix protein 1 (M1) and polymerase PB1 (PB1) from IFV-A; nucleoprotein (NP), nonstructural protein 1 (NS1) and polymerase PA (PA) from IFV-B; 5'Untranslated Region (5'UTR) from HRV; RNA-dependent RNA polymerase (L) and fusion glycoprotein (F) from RSV; DNA polymerase (E2B) from HAdV; and Deoxyribon uclease (DNase) and MgpC family cytadherence protein (mgpC) from MP (Table 1). BLAST analysis revealed that all the primers and probes had good specificity and did not cause the amplification of genes from other pathogens.

**Fig 1:**
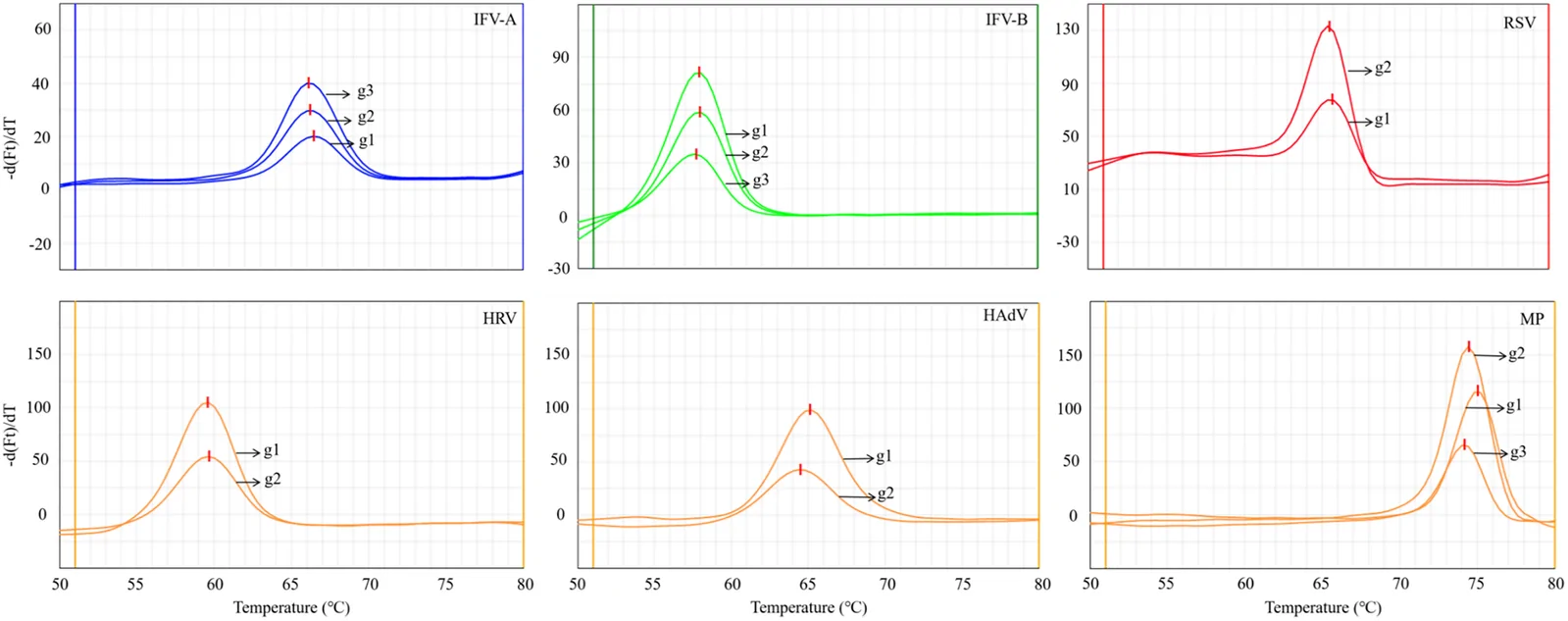
Comparison of melting curve peaks (Rm) in the mqPCR-PMC assay using various groups (g1, g2, g3) of primer and probe for six pathogens. The sequences of primers and probes were listed in Table 1. The probes targeting IFV-A was labeled with 6-carboxyfluorescein (blue line); the probes targeting IFV-B was labeled with hexachloro-6-methylfluorescein (green line); the probes targeting RSV was labeled with indodicarbocyanine-5 (red line); the probes targeting HRV, HAdV and MP were labeled with carboxy-X-rhodamine (orange line).

**Fig 2:**
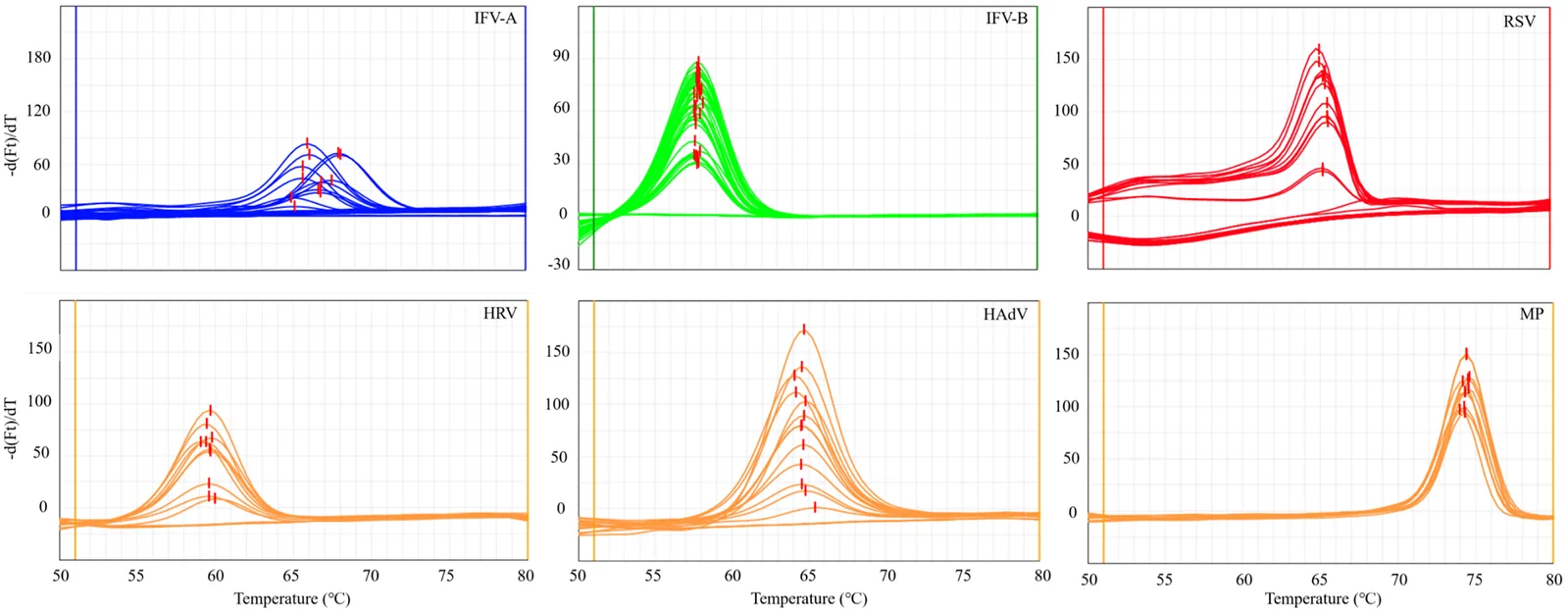
Comparison of melting curve peaks (Rm) in mqPCR-PMC assay using various concentrations of primer and probe for six pathogens. The concentration of forward and reversed primers ranged from 40 to 105 nM and 250 to 490 nM, under ratios of 1/5, 1/6, and 1/7, while the concentration of probes ranged from 40 to 105 nM. The probes targeting IFV-A was labeled with 6-carboxyfluorescein (blue line); the probes targeting IFV-B was labeled with hexachloro-6-methylfluorescein (green line); the probes targeting RSV was labeled with indodicarbocyanine-5 (red line); the probes targeting HRV, HAdV and MP were labeled with carboxy-X-rhodamine (orange line).

When recombinant plasmids containing target fragments were used as templates, the mqPCR-PMC assay results revealed that different groups of primers and probes led to differences in the melting curves (Fig 1), and the group with the maximum melting curve peak area was selected: g3 for the PB1 gene from IFV-A, g1 for the NP gene from IFV-B, g2 for the F gene from RSV, g1 for the 5’ UTR from HRV, g1 for the E2B gene from HAdV, and g2 for the DNase gene from MP. In the optimal group, the downstream primers might have higher Tm values and less possibility to form secondary structure, or the gene templates might have lower GC % content and no hairpin structures or G-quadruplexes. These factors facilitated the binding of primers to templates and the polymerase extension, resulting in more obvious melting peak in the -dF/dT curves. Subsequently, different concentration ratios of the optimized primers and probes were prepared and analyzed. Among them, the maximum Rm values were obtained at concentrations of 60, 420, and 72 nM for the forward and reverse primers and the probes, respectively (Fig 2).

**Fig 3:**
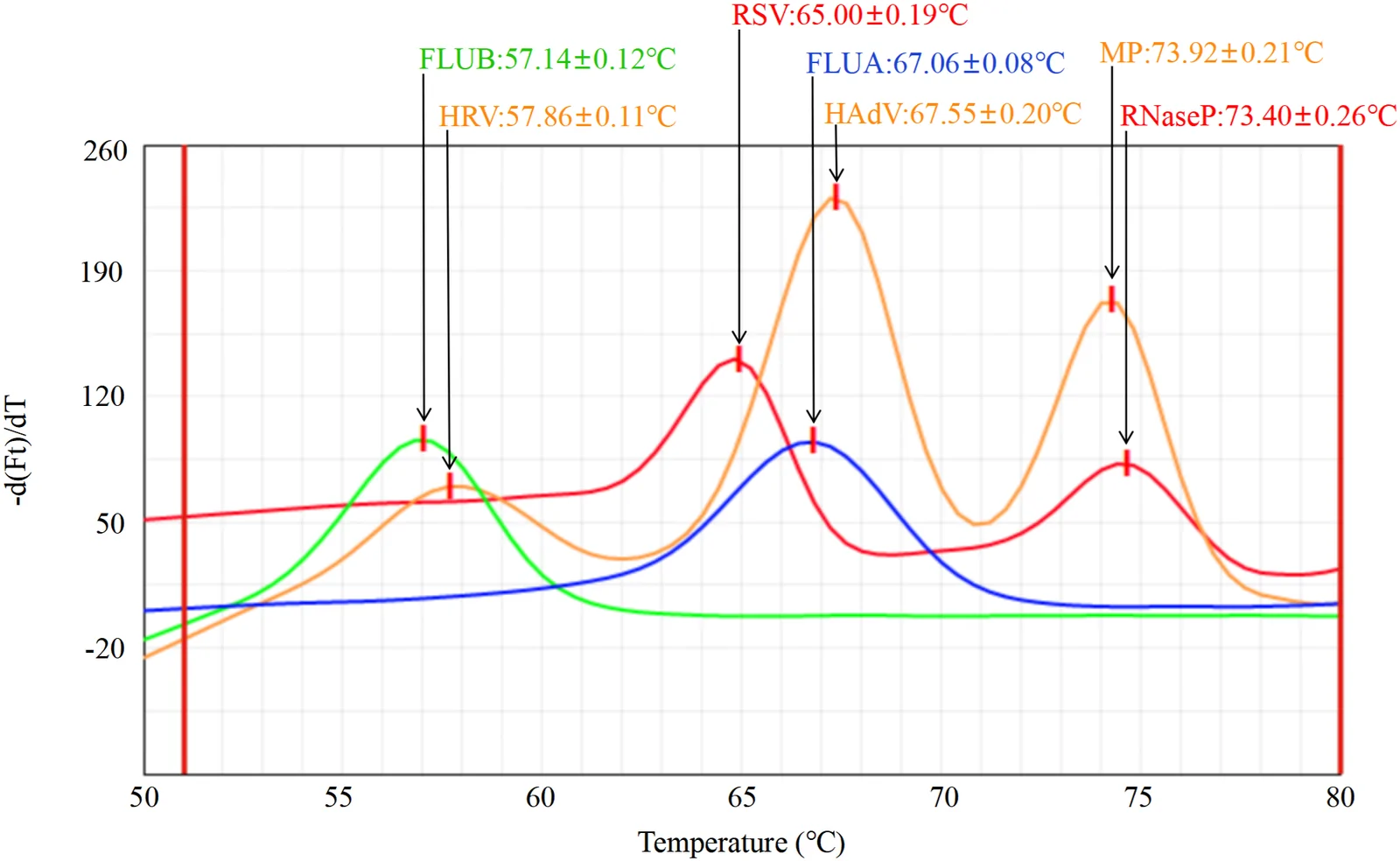
Melt curve analysis showing the melting temperature (Tm) peaks of six pathogens and IC. The probes targeting IFV-A was labeled with 6-carboxyfluorescein (blue line); the probes targeting IFV-B was labeled with hexachloro-6-methylfluorescein (green line); the probes targeting RSV was labeled with indodicarbocyanine-5 (red line); the probes targeting HRV, HAdV and MP were labeled with carboxy-X-rhodamine (orange line).

Finally, the optimized mqPCR-PMC system was verified using the positive control as the amplification template. All primer and probe pairs successfully amplified the corresponding target genes, generating melting curves with a single peak. The equivalent Tms of each pathogen were 67.06 ± 0.08 ℃ (IFV-A), 57.14 ± 0.12 ℃ (IFV-B), 57.86 ± 0.11 ℃ (HRV), 67.55 ± 0.20 ℃ (HAdV), 73.92 ± 0.21 ℃ (MP), 65.00 ± 0.19 ℃ (RSV) and 74.40 ± 0.26 ℃ (RNaseP) (Fig 3). The software automatically interpreted the Tm value and the corresponding Rm value for each melting peak on the result analysis interface. And the minimum Tm separation achieved within a channel was 1 ℃. According to the predefined Tm, the positive target pathogen could be identified and the potential peak overlap or interference could be excluded. The turnaround time from nucleic acid extraction to the final MCA results was estimated to be approximately 2.5 hours.

### Specificity, sensitivity and repeatability of the mqPCR-PMC assay

The specificity results of the mqPCR-PMC assay are summarized in Table 2. All the detection results were consistent with the theoretical results. For the 20 positive substances, every target was identified correctly. For the 37 negative substances, no amplification and melting signals were detected, demonstrating no cross-reactivity among non-target species.

**Fig 4:**
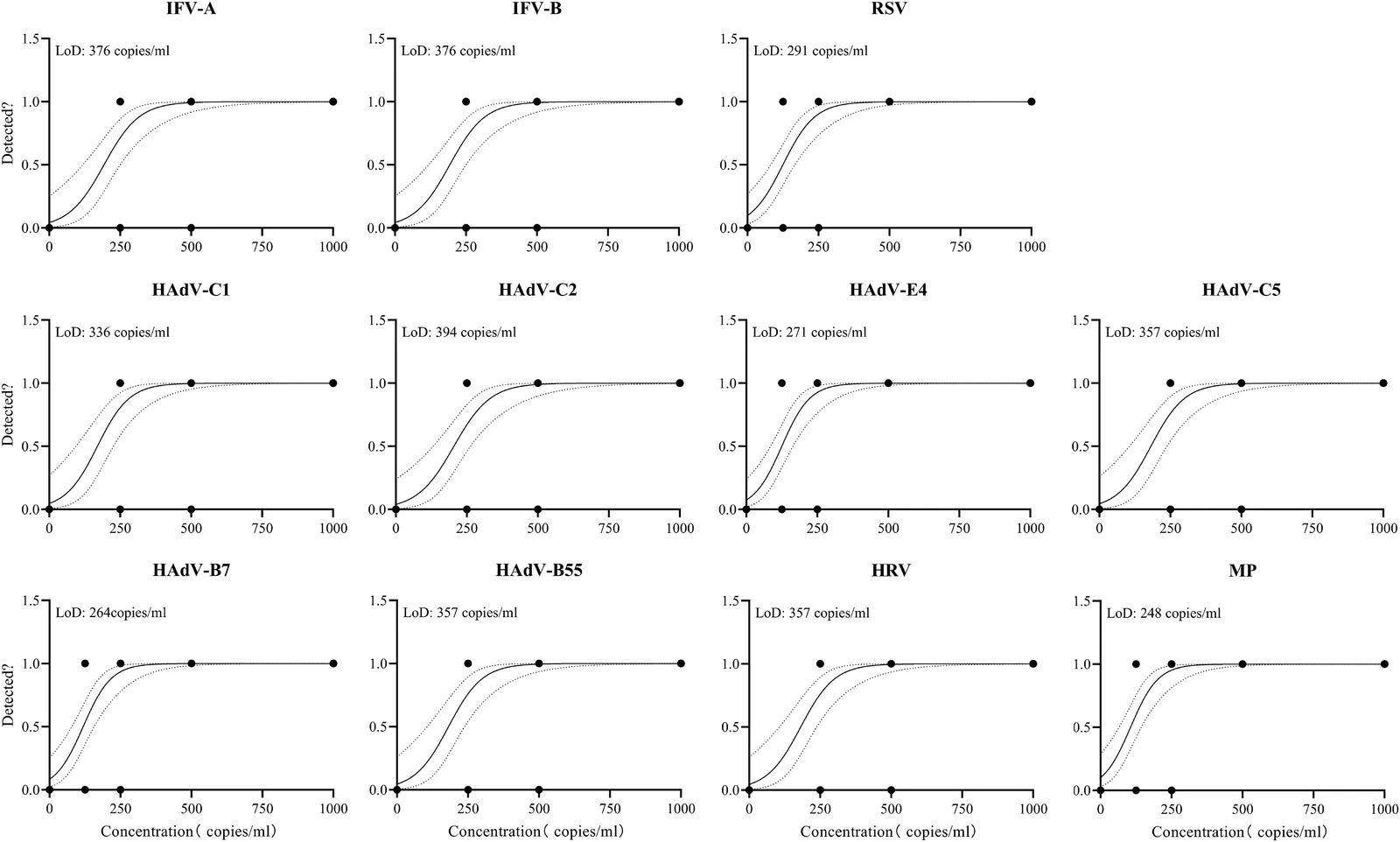
Simple logistic regression analysis results for the mqPCR-PMC assay. Simple logistic regression analysis was performed using GraphPad Prism 10 software. The LoDs for six targets were 376 copies/ml (IFV-A), 376 copies/ml (IFV-B), 291 copies/ml (RSV), 264-394 copies/ml (HAdV), 357 copies/ml (HRV), and 248 copies/ml (MP), respectively.

The sensitivity of the mqPCR-PMC assay was determined using dilutions of the nucleic acid RM. The detection rates at different concentrations are shown in Table 3. Addional dilutions testing of 125 copies/ml were performed on E4 and B7 subtypes of HAdV, MP and RSV, due to the perfect separation error appearing in the simple logistic regression analysis. Among the six pathogens, MP had the highest sensitivity, with the LoD of 248 copies/ml. And this was followed by RSV (291 copies /ml) and HRV (357 copies /ml). The LoDs of IFV-A and IFV-B were both 376 copies/ml. The sensitivity for HAdV differed across subtypes, ranging from 264 copies /ml for B7 to 394 copies /ml for C2 (Fig 4). The difference in sensitivity might be attributed to the difference in binding efficiency between the primers and templates. For example, an online silico analysis (OligoEvaluator™ - Sequence Analysis) revealed that the primer sequence matching with subtype B7, had a Tm value of 64.7 ℃, larger than that of subtype C2 (62.1 ℃), and the primer for subtype B7 had a GC % of 61.1%, lower than that of subtype C1 (66.7%). Larger Tm and lower GC % might facilitate the binding stability of primers and templates and polymerase extension.

**Table 3:** Limit-of-detection results for the mqPCR-PMC assay.

	No. detected/No. replicates (%) at each concentration (copies/ml)					
	2000	1000	500	250	125a	0
**IFV-A**	20/20(100)	20/20(100)	19/20(95)	16/20(80)	/	0/20(0)
**IFV-B**	20/20(100)	20/20(100)	19/20(95)	16/20(80)	/	0/20(0)
**HRV**	20/20(100)	20/20(100)	19/20(95)	17/20(85)	/	0/20(0)
**HAdV-C1**	20/20(100)	20/20(100)	19/20(95)	18/20(90)	/	0/20(0)
**HAdV-C2**	20/20(100)	20/20(100)	19/20(95)	15/20(75)	/	0/20(0)
**HAdV-E4**	20/20(100)	20/20(100)	20/20(100)	17/20(85)	13/20(65)	0/20(0)
**HAdV-C5**	20/20(100)	20/20(100)	19/20(95)	17/20(85)	/	0/20(0)
**HAdV-B7**	20/20(100)	20/20(100)	20/20(100)	17/20(85)	14/20(70)	0/20(0)
**HAdV-B55**	20/20(100)	20/20(100)	19/20(95)	17/20(85)	/	0/20(0)
**MP**	20/20(100)	20/20(100)	20/20(100)	17/20(85)	16/20(80)	0/20(0)
**RSV**	20/20(100)	20/20(100)	20/20(100)	16/20(80)	14/20(70)	0/20(0)

^a^/means not detect

In the repeatability experiment, the CVs for Tm were 0.11% (IFV-A), 0.21% (IFV-B), 0.20% (HRV), 0.29% (HAdV), 0.28% (MP) and 0.30% (RSV). And the CVs for Rm were 3.56% (IFV-A), 1.96% (IFV-B), 3.26% (HRV), 4.10% (HAdV), 1.59% (MP) and 3.99% (RSV).

### Clinical validation of the mqPCR-PMC assay

**Fig 5:**
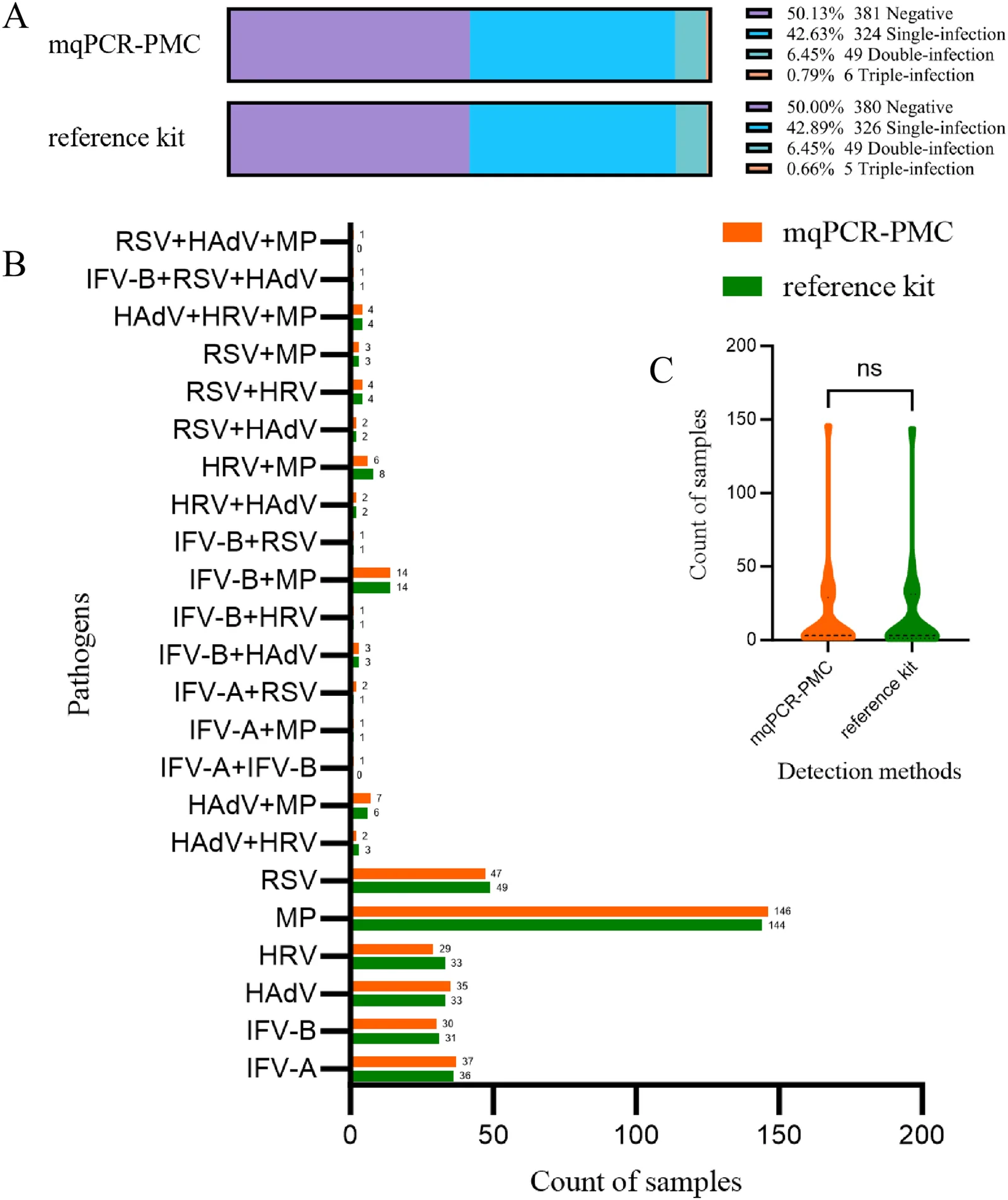
The comparison between two methods in detected pathogen species. (A) The detection rates and count of negative, single-, double- and triple-infections; (B) the count of samples for different pathogens; (C) The method-wise statistics of the pathogen results, ns means no significant difference was found by Wilcoxon test (P = 0.7603, data from (B)).

A total of 760 pharyngeal samples (380 positive and 380 negative) were analyzed to verify the clinical performance of the mqPCR-PMC assay. The results of the mqPCR-PMC assay were analyzed using a commercial kit as a reference. The turnaround time was one hour longer than that of the commercial kit (1.5 hour). The pathogen species detected in the positive samples are listed in Fig 5. The reference kit showed that 326 samples (42.89%) were positive for one single pathogen, and 54 samples (7.11%) were positive for more than one pathogen. Compared to the reference kit, the mqPCR-PMC assay detected two less samples positive for single infection (324, 42.63%) and one more samples positive for co-infections (54, 7.24%) (Fig 5A). Slight differences were detected for six single-infection, five double-infection and one triple-infection (Fig 5B), but no significant difference was found in general between the two methods (P-value = 0.7603, Wilcoxon test)(Fig 5C). The sensitivity, specificity, PPA, and NPA with 95% CIs, as well as the kappa value for each pathogen, are summarized in Table 4. The NPAs of the mqPCR-PMC assay exceed 97% for all six pathogens, and the PPAs exceed 98%, except for HRV (87.3%). The kappa values ranged from 0.927 to 1.000, indicating almost perfect agreement between the two methods for all six pathogens. In particular, the sensitivities of the mqPCR-PMC assay versus qPCR were 100% for IFV-A, IFV-B and HAdV, and the specificities were 100% for both IFV-B and HRV. The NPVs of the mqPCR-PMC assay exceed 99% for all six pathogens, and the PPVs exceed 92% (Table 5).

**Table 4:** Positive and negative agreement between the mqPCR-PMC and real-time RT-PCR assay.

Pathogens	mqPCR-PMC	Commercial kit		%Sensitivity(95% CI)	%Specificity(95% CI)	%PPA(95% CI)	%NPA(95% CI)	Kappa^a^(95% CI)
		Positive	Negative					
IFV-A	Positive	38	3	100 (90.8-100)	99.58 (98.79-99.89)	100 (90.8-100)	99.6 (98.8-99.9)	0.960 (0.915-1.99)
	Negative	0	719					
IFV-B	Positive	51	0	100 (93.0-100)	100 (99.46-100)	100 (93.0-100)	100 (99.5-100)	1.000 (1.00-1.00)
	Negative	0	709					
HRV	Positive	48	0	87.27 (76.0-93.7)	100 (99.46-100)	87.3 (76.0-93.7)	100 (99.5-100)	0.927 (0.874-0.981)
	Negative	7	705					
HAdV	Positive	54	3	100 (93.4-100)	99.58 (98.769-99.88)	100 (93.4-100)	99.6 (98.8-99.9)	0.971 (0.938-1.000)
	Negative	0	703					
MP	Positive	169	13	99.41 (96.7-99.97)	97.80 (96.27-98.71)	99.4 (96.7-100)	97.8 (96.3-98.7)	0.948 (0.921-0.975)
	Negative	1	577					
RSV	Positive	60	1	98.36 (91.3-99.9)	99.86 (99.19-99.99)	98.4 (91.3-99.9)	99.9 (99.2-100)	0.982 (0.958-1.000)
	Negative	1	698					

^a^Kappa < 0: No agreement; Kappa between 0.00 and 0.20: Slight agreement; Kappa between 0.21 and 0.40: Fair agreement; Kappa between 0.41 and 0.60: Moderate agreement; Kappa between 0.61 and 0.80: Substantial agreement; Kappa between 0.81 and 1.00: Almost perfect agreement.

**Table 5:** Positive and negative predictive values of the mqPCR-PMC assay.

	IFV-A	IFV-B	HRV	HAdV	MP	RSV
%PPV(95%CI)	92.7(80.6-97.5)	100(93.0-100)	100(92.6-100)	94.7(85.6-98.6)	92.9(88.2-95.8)	98.4(91.3-99.9)
%NPV(95%CI)	100(99.5-100)	100(99.5-100)	99.0(98.0-99.5)	100(99.5-100)	99.8(99.0-100)	99.9(99.2-100)

Seven positive HRV samples were determined to be negative by the mqPCR-PMC assay. The sequencing results revealed that they were enteroviruses. They were further successfully amplified by an enterovirus nucleic acid detection kit (Sansure, Hunan, China). This difference might be due to the non-specific amplification of the reference kit. Moreover, 20 samples that tested positive for IFV-A (3), HAdV (3), MP (13) and RSV (1) using the assay, were considered negative by the reference kit. Sequencing indicated that these samples were indeed positive. These results indicated that the sensitivity of the assay was greater than that of the reference kit.

## Discussion

During the COVID-19 pandemic of 2020, the implementation of non-pharmaceutical interventions (NPIs) clearly reduced the global detection rate of respiratory pathogens, especially influenza virus and RSV [28–30]. Following the relaxation of NPIs, some respiratory pathogens have reemerged globally in many countries. The occurrence of RSV increased 25- to 50-fold compared with that in previous pre-pandemic years, and the prevalence of rhinovirus and enterovirus infections increased approximately 16-fold [31]. In Henan, China, the numbers of positive cases and the rates of influenza virus and RSV infections in children increased significantly after the cessation of NPI measures in December 2022 [32]. Beginning in 2023, the reemergence of MP was identified as an epidemic in four UN regions, with reports indicating increased hospitalizations and ICU admissions for both adults and children [33]. In the summer of 2023, an increasing trend of MP infections occurred among children in China [34]. Therefore, continuous surveillance of respiratory pathogens is still necessary to aid in diagnosis and treatment.

Several studies have reported melting curve strategies for the detection of infectious pathogens. Tavares et al. developed a melting-curve-based multiplex real-time PCR assay for the detection of four respiratory viruses [35]. Otaguiri et al. reported a melting-curve-based multiplex real-time PCR assay for the simultaneous detection of *Streptococcus agalactiae* and its resistance genes [36]. Ma et al. evaluated the performance of a dual-gene qPCR melting curve assay (DGPMC) in detecting tuberculosis among patients with suspected pulmonary tuberculosis [37]. In these studies, the process of double-stranded DNA unwinding was monitored through the release of embedded fluorescent dyes, such as SYBR Green. In contrast, specific TaqMan probes were used in this study. In addition to the advantage of higher specificity, the Tm values of the pathogens varied over a wider rangedepending on the length of the probe sequence rather than the amplification product. Additionally, more observation channels with different fluorescence could be used. These factors facilitate designs that distinguish more pathogens.

The primer and probe sequences used in our study differed from those commonly used in previously reported papers. On the one hand, we could obtain the most suitable Tm values to distinguish different pathogens by adjusting the sequences and positions of the probes. For instance, the maximum melting curve peak area appeared when the polymerase PB1 gene was used as the amplification template rather than the commonly used matrix protein 1(M1) gene [38]. For RSV, the RNA-dependent RNA polymerase (L) and fusion glycoprotein (F) genes were used for primer and probe design, whereas the nucleoprotein (N) gene was used by Sanghavi et al. or Feng et al. [39,40]. In addition, three pairs of primers were selected to amplify subtypes A, B, and C of HRV, with the goal of avoiding the non-specific amplification of enteroviruses. These primers and probes have been proven to have good sensitivity and specificity in the detection of clinical samples in our study. To further compare the two methods, four random weakly positive samples (Ct > 30) of IFV-A, HAdV, MP, and RSV were diluted 10- or 20-fold and detected in five replicates. The positive rate of the mqPCR-PMC assay was higher than that of the reference kit (S2 Fig). On the other hand, we could select primers with the highest binding efficiency, thus enhancing the detection sensitivity. The LoD of this assay ranged from 248 to 394 copies/ml, whereas it ranged from 5,000–500,000 copies/ml in Liao’s research, which was also based on a melting curve strategy [41].

Besides the advantage in sensitivity, the mqPCR-PMC assay was more cost-effective than the reference method. The obvious cost difference lies in the number of amplification tubes and reagent volume. The former required one amplification tube filled with 50 ul of reaction system per sample, but the latter doubled to two tubes, each containing 45 ul of reaction system. The single tube detection style would be very meaningful for cost savings, particularly in the large-scale detection or resource-limited settings.

In addition, other multiplex PCR platforms such as BioFire FilmArray Respiratory Panel or Luminex NxTAG were also widely used for FRS detection. Compared with them, the mqPCR-PMC assay had lower cost due to good instrument compatibility and low reagent cost. This assay could be carried out on most qPCR instruments, no needing for special cassette instrument or flow hybridization instrument. And it used conventional reagents and consumables, no needing for microfluidic card or hybrid microspheres. Although the BioFire FilmArray exhibited higher sensitivity and shorter detection time (about 1 hour) basing on nested qPCR and High Resolution Melting (HRM) [42], it could detect one sample per experiment. while the mqPCR-PMC assay had greater sample throughput (96 samples per experiment) with high sensitivity. basing on the different encoded fluorescent microspheres and TAG sequence tags, Luminex NxTAG had higher throughput in both of pathogen targets and samples but longer detection time (about 5 hours). And it was reported that the clinical consistency is slightly lower in detecting some pathogens, for example, the NxTAG Respiratory Pathogen Panel v2 assay had consistency of only 92.3% and 90.3% for MP and parainfluenza 4 with Sanger sequencing [43].

This study had two limitations. i) Due to the small number of clinical samples and the preferential sampling strategy, the pathogen positive rate in this paper cannot reflect the natural prevalence of these pathogens in the clinical respiratory infection population. ii) Using the reference kit, which had produced false positive/negative results, to evaluate the sensitivity, specificity, PPV and NPV of the mqPCR-PMC assay, might lead to a non-objective result. A validation cohort with more samples and increasing comparisons with other multiplex nucleic acid testing techniques such as t/mNGS sequencing would be implemented in the future.

## Conclusions

In summary, we developed an mqPCR-PMC assay to simultaneously detect six respiratory pathogens, namely, IFV-A, IFV-B, RSV, HRV, HAdV, and MP, in one tube. It was more accurate for the detection of HRV genes than the reference kit, which utilized fluorescent qPCR technology. It showed better sensitivity than the reference kit and previously published methods. Relying on variations in the fluorescent groups and Tm values of the probes provides a strategy for simultaneously detecting more pathogen targets in one tube. We are trying to construct a reaction system that includes other viruses and bacteria, such as SARS-CoV-2, human parainfluenza virus, and *Bordetella pertussis*. Because of its high reliability and low LoD, this assay will be very helpful for the diagnosis of respiratory infectious diseases.

## Supporting information

S1 Fig The Rm distributions to determine the cutoff value of an effective melting peak. Rm ≥ 5 was used as the cutoff value of an effective melting peak, basing on the distributions of negative controls and non target samples. The negative group was include 37 negative quality control and 10 negative samples, with the Rm values ranged from 0 to 4.890. The positive group was include 50 random positive nucleic acid reference materials at the LoD concentration, with the Rm values ranged from 10.88 to 67.80. There was a significant difference between the two groups (Welch t test, *p* < 0.001).
(TIF)

S2 Fig Comparison of the positive detection rate between two methods. In the mqPCR-PMC assay, target IFV-A was labeled with blue line, HAdV and MP were labeled with orange line, and RSV and IC was labeled with red line at 65 ℃ and 73.4 ℃. The positive detection rate for the four targets were all 5/5. In the reference kit analysis, target IFV-A was labeled with blue line, MP and RSV were labeled with red line, and IC was labeled with orange line. The positive detection rate for the four targets were 1/5, 0/5, 1/5 and 2/5.
(TIF)

S1 Table Concentrations of primers and probes used in triple factors orthogonal test. (PDF)
