# Supplementary material for: Development of a multiplex real-time PCR assay with fluorescence probe-melting-curve analysis for one-tube detection of respiratory pathogens
Source: PLoS One. 2026 Sep 9;21(9):e0357377. doi: 10.1371/journal.pone.0357377 (PMC13557345; doi:10.1371/journal.pone.0357377)
Supplement: Supplementary file 1 [file pone.0357377.s003.pdf]

S1 Table. Concentrations of primers and probes used in riple factors orthogonal test.

Exparimental tube	Forward primer (nM)	Reversed primer (nM)	Probe (nM)
**1**	50	250	40
**2**	60	300	48
**3**	70	350	56
**4**	50	300	40
**5**	60	360	48
**6**	70	420	56
**7**	50	350	40
**8**	60	420	48
**9**	70	490	56
**10**	50	250	50
**11**	60	300	60
**12**	70	350	70
**13**	50	300	50
**14**	60	360	60
**15**	70	420	70
**16**	50	350	50
**17**	60	420	60
**18**	70	490	70
**19**	50	250	60
**20**	60	300	72
**21**	70	350	84
**22**	50	300	60
**23**	60	360	72
**24**	70	420	84
**25**	50	350	60
**26**	60	420	72
**27**	70	490	84
**28**	50	250	75
**29**	60	300	90
**30**	70	350	105
**31**	50	300	75
**32**	60	360	90
**33**	70	420	105
**34**	50	350	75
**35**	60	420	90
**36**	70	490	105
